# Intussusception among infants in Tanzania: findings from prospective hospital-based surveillance, 2013-2016

**DOI:** 10.11604/pamj.supp.2021.39.1.21358

**Published:** 2021-07-28

**Authors:** Mwajabu Mbaga, David Msuya, Lazaro Mboma, Bhavin Jani, Fausta Michael, Christopher Kamugisha, Said Ali Said, Abdulhamid Saleh, Jason Mathiu Mwenda, Margaret Cortese

**Affiliations:** 1 Muhimbili National Hospital, Dar Es Salaam, United Republic of Tanzania,; 2 Kilimanjaro Christian Medical Centre, Moshi, United Republic of Tanzania,; 3 Mbeya Zonal Referral Hospital, Mbeya, United Republic of Tanzania,; 4 World Health Organization, Country Office, Dar Es Salaam, United Republic of Tanzania,; 5 Ministry of Health, Community Development, Gender, Elderly and Children, Dar Es Salaam, United Republic of Tanzania,; 6 Mnazi Mmoja Hospital, Zanzibar, United Republic of Tanzania,; 7 Immunization Program, Ministry of Health, Zanzibar, United Republic of Tanzania,; 8 World Health Organization, Regional Office for Africa, Brazzaville, Republic of Congo,; 9 Division of Viral Diseases, National Center for Immunization and Respiratory Diseases, Centers for Disease Control and Prevention, Atlanta, Georgia, United States of America

**Keywords:** Intussusception, infants, Tanzania

## Abstract

**Introduction:**

intussusception surveillance was initiated in Tanzania in 2013 after monovalent rotavirus vaccine was introduced, as part of the 7-country African evaluation to assess whether the vaccine was associated with an increased risk of intussusception. An increased risk from vaccine was not identified. Published data on intussusception in Tanzanian infants are limited.

**Methods:**

prospective intussusception surveillance was conducted at 7 referral hospitals during 2013-2016 to identify all infants with intussusception meeting Brighton Level 1 criteria. Demographic, household and clinical data were collected by hospital clinicians and analyzed.

**Results:**

a total of 207 intussusception cases were identified. The median age of cases was 5.8 months and nearly three-quarters were aged 4-7 months. Median number of days from symptom onset to admission at treatment hospital was 3 (IQR 2-5). Seventy-eight percent (152/195) of cases had been admitted at another hospital before transfer to the treating hospital. Enema reduction was not available; all infants were treated surgically and 55% (114/207) had intestinal resection. The overall case-fatality rate was 30% (62/206). Compared with infants who survived, those who died had longer duration of symptoms before admission to treatment hospital (median 4 vs 3 days; p < 0.01), higher rate of intestinal resection (81% [60/82] vs 44% [64/144], p < 0.001), and from families with lower incomes (i.e., less likely to own a television [p < 0.01] and refrigerator [p < 0.05).

**Conclusion:**

Tanzanian infants who develop intussusception have a high case-fatality rate. Raising the index of suspicion among healthcare providers, allocating resources to allow wider availability of abdominal ultrasound for earlier diagnosis, and training teams in ultrasound-guided enema reduction techniques used in other African countries could reduce the fatality rate.

## Introduction

Intussusception is a medical emergency involving obstruction of the intestine. It can be fatal if not timely treated. The highest incidence rates are in infants. The mean incidence of intussusception has been estimated as 74 per 100,000 (range: 9-328) and peak incidence occurs among infants 5-7 months of age [1, 2]. Intussusception incidence also varies by ethnic group and geographical area. For most cases in infants, the cause of the intussusception is not known.

In 1999, the first licensed rotavirus vaccine, RotaShield, was introduced to reduce the incidence of severe gastroenteritis caused by rotavirus but then was withdrawn from the market due to an association with intussusception [3]. Based on the facts that rotavirus is the most common cause of severe, dehydrating gastroenteritis among children globally, resulting in approximately 400,000 deaths each year, in 2006, two rotavirus vaccines RotaTeq and Rotarix were licensed and subsequently introduced in childhood immunization programs. Many countries which introduced these new vaccines opted to conduct post-marketing surveillance for intussusception.

In Tanzania, before vaccine introduction, rotavirus was the most common cause of acute diarrhea, causing an estimated 40% of acute diarrhea hospitalizations among children < 5 years of age [4]. The country introduced monovalent rotavirus vaccine (RV1, Rotarix, GlaxoSmithKline) in January 2013. Tanzania was one of 7 countries in Africa that conducted intussusception surveillance following introduction of RV1 to evaluate the association between the vaccine and intussusception, using a self-controlled case series design. An increased risk of intussusception following RV1 was not identified in that comprehensive study [5]. In this current report, we present additional data on the intussusception cases enrolled in Tanzania.

## Methods

### Population and study settings

Tanzania began intussusception surveillance in infants as part of rotavirus vaccine safety monitoring in 2013. In that year, the country had a total of 1.9 million births. A prospective study was conducted in seven referral hospitals that provide pediatric surgical services. These included Muhimbili National Hospital in Dar es Salaam region, Kilimanjaro Christian Medical Centre in Kilimanjaro region Mbeya Zonal Referral Hospital in Mbeya region, Bugando Medical Centre and Sekou Toure Hospital in Mwanza region, Bombo Regional Referral Hospital in Tanga region, and Mnazi Mmoja Hospital in Zanzibar. These were the main hospitals capable of managing children with intussusception. During the surveillance period, intussusception reduction by enema had not been implemented at these hospitals and all cases were treated surgically. Pediatric surgeons and nurses working in surgical departments from these hospitals were oriented in the surveillance procedures and tools. These hospitals were also involved in the surveillance of acute gastroenteritis caused by rotavirus. The evaluation was deemed public health nonresearch during the human subjects review at the Centers for Disease Control and Prevention, and received an exemption by the WHO Research Ethics Review Committee [5].

### Data sources

A standard case form was used to capture variables. These include demographic information: age, gender, date of symptoms onset, admission date, diagnosis and treatment method and outcome. Feeding and household characteristics were also collected, including ownership of certain items as a reflection of socioeconomic status. Information on receipt of rotavirus vaccine was collected and used in the self-controlled case series, and is not included in this report. Data on laboratory-confirmed rotavirus gastroenteritis among infants were obtained from the active surveillance system conducted at the same or nearby hospitals as those performing intussusception surveillance. Data on rotavirus disease during 2014 and 2015 were used because infants were consistently enrolled into rotavirus surveillance during these years. For this comparison, data from Zanzibar and Tanga were excluded (the former because of very large numbers of infants enrolled in the rotavirus surveillance relative to the other sites, and Tanga because no intussusception cases were enrolled after 2013).

### Case definition

A case of intussusception was defined as child < 12 months of age at diagnosis meeting the Brighton Collaboration criteria for level 1 of diagnostic certainty for intussusception [6]. Level 1 of diagnostic certainty requires confirmation of intussusception during surgery or by specific radiological findings if reduction occurred by enema, or at autopsy. Only first episodes of intussusception were enrolled into surveillance.

### Statistical analysis

The demographic, household and clinical characteristics of case-patients from all hospitals were summarized using descriptive statistics. For 5 patients, date of birth was not available to calculate exact age so age in months was imputed as the reported age in months plus 0.5. Characteristics of infants who survived were compared with those who died in univariate analyses using Wilcoxon rank-sum test for continuous variables, and chi-square for categorical variables. Analyses were performed using Epi-Info 3.5.1 (EpiInfo, GA, USA), Microsoft Excel 2007 (Microsoft®, WA, USA), and Stata 13 (College Station, TX).

**Needs disclaimer:** the findings and conclusions in this paper are those of the authors and do not necessarily represent the official position of the Centers for Disease Control and Prevention or the World Health Organization.

## Results

**Figure 1:**
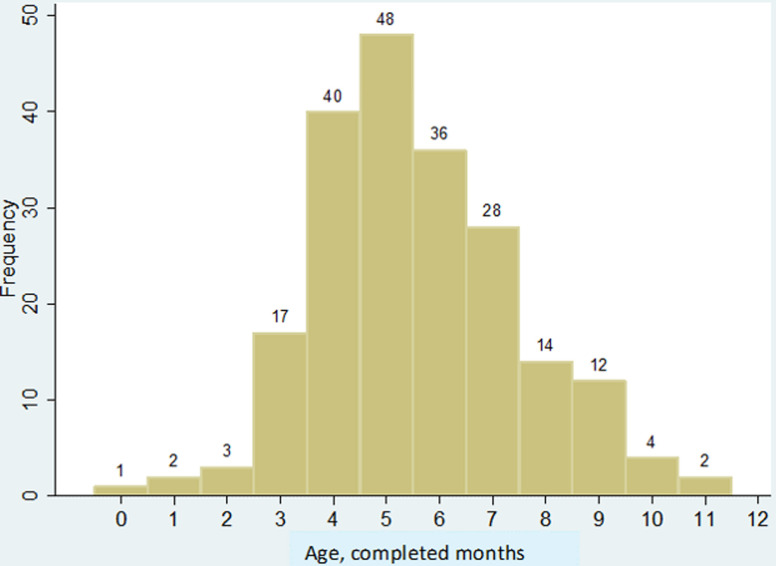
number of intussusception cases among infants, by age at symptom onset (age in completed months)

A total of 207 children < 12 months of age with intussusception were enrolled during the four years, January 2013 - December 2016. Males were predominant at 61% (126/207). The median age at symptom onset was 5.8 months (IQR 4.8-7.3, range 2 days-11.7 months), and 73% of the intussusception cases occurred at ages 4 through 7 months (Figure 1). Among those for whom information was provided, (> 82% response, depending on variable), 96% (190/197) of infants had been breastfed for some period, 27% (46/169) had not yet started taking another type of milk daily (other than breastmilk) and 28% (48/169) had not yet started taking solid foods each day. Of the cases´ households, the median number of persons in the residence was 5 (range 2-16), and 31% (56/181) reported that someone in the household was employed. Sixty percent (106/176) reported having electricity usually for most hours of the day (31% [54/176] had no electricity and 9% [16/176] had electricity for some hours), 93% (176/190) of families had a telephone, 56% (107/190) had a television, 15% (28/190) had a motorcycle and 8% (15/189) had a car.

Of the 195 infants for which such information was available, 78% (152/195) of infants had been hospitalized at another hospital before arriving at the treatment hospital. The overall median number of days from symptom onset to admission at the treatment hospital was 3 (IQR 2-5; range 0-17 days). The number of days from symptom onset to admission at the treatment hospital was greater among those who had been transferred to the treatment hospital (median 4 days) than among those whose first admission was at the treatment hospital (median 2 days, p = 0.005); the number of days from symptom onset to admission at the first hospital tended to be shorter among those who were transferred (median 1 day) than those who were never transferred (median 2 days, p = 0.056). Infants were transferred a median of 1 day (IQR 1-3, range 0-9) after admission at the first hospital.

Sixty-eight percent (139/205) of infants had had an abdominal ultrasound during their evaluation. All the cases were surgically treated, and 55% (114/207) had intestinal resection. Overall, 30% of infants died (62/206; one additional infant survived surgery without resection and was transferred to another hospital but ultimate outcome was not available). Fatality rate was similar across the hospitals (excluded hospitals with 6 or fewer total case). Among the infants who did not undergo intestinal resection, the fatality rate was 13% (12/92) while the fatality rate was 44% (50/114) among those who had resection (p < 0.0001). Of those who died, death occurred a median of 2 days (IQR 1-4, range 0-21) after admission to the treatment hospital. Among those surviving, the median duration of hospitalization was 6 days (IQR 4-9; range 2-47 days) with a median duration of 5 days for those without resection and 8 days for those with resection (p < 0.0001).

**Figure 2:**
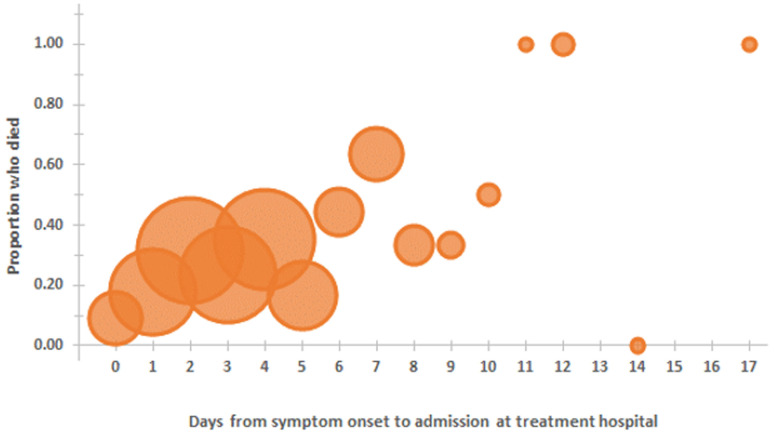
proportion of infants who died, by days from symptom onset to admission at treatment hospital (size of circle is proportionate to the number of infants)

**Figure 3:**
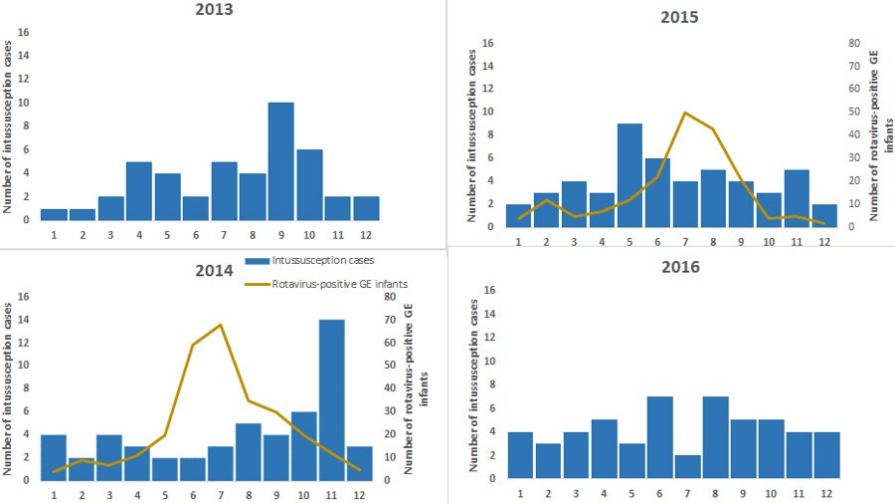
number of intussusception cases by calendar month 2013-2016, and seasonality of rotavirus diarrhea, 2014-2015 (number of Infants with rotavirus diarrhea detected in surveillance

Among the variables available and compared, some differences were identified between infants who died and those who survived (Table 1). Households of infants who died were less likely to have a television or refrigerator. As expected, a greater proportion of infants who died had had resection. The number of days from symptom onset to admission at the treatment hospital was greater among infants who died (median 4 days, IQR 2-6, range 0-17) compared with those who survived (median 3 days, IQR 2-4, range 0-14). There was a trend toward increased probability of death with each additional day of time from symptom onset to admission at treatment hospital (Figure 2), but this was found to only be true for infants whose households had a television and not true for infants whose households did not have a television (data not shown) so regression modeling to estimate this increased risk per additional symptomatic day was not further pursued. Intussusception cases occurred throughout the year (Figure 3), without clear pattern or evidence of seasonality. There was no evidence of greater proportion of intussusception cases occurring during peak months of rotavirus disease.

**Table 1:** characteristics of infants with intussusception, by outcome (died vs survived)#

Characteristic	Among those who died, N=62 (# yes/total)	% yes	Among those who survived, N=144 (# yes/total)	% yes	p-value
Male gender	35/62	56%	90/144	62%	0.42
Age at symptom onset (months), median	5.8		5.8		0.81
Birthweight (kg), median	3.1		3.0		0.34
Number of household members, median	5		4		0.29
At least one household/member is employed	11/50	22%	45/130	35%	0.10
Household has electricity/usually for most hours of day	26/47	55%	80/129	62%	0.42
House has 3 or more rooms	27/48	56%	63/129	49%	0.38
**Family owns**					
telephone	48/51	94%	127/138	92%	0.63
refrigerator	12/51	23%	60/138	43%	0.012
bicycle	19/51	37%	46/138	33%	0.61
motorcycle	9/51	18%	19/138	14%	0.51
car	2/51	4%	13/137	9%	0.21
television	20/51	39%	87/138	63%	0.003
**Clinical features**					
Days from symptom onset to admission at treatment hospital, median (IQR; range)	4 (2-6; 0-17)		3 (2-4; 0-14)		0.005
Had been admitted at another/facility before treatment hospital	47/59	80%	105/135	78%	0.77
Had abdominal ultrasound	41/62	66%	98/142	69%	0.68
Had intestinal resection	50/62	81%	64/144	44%	<.001

* excludes one case with unknown outcome

## Discussion

Our surveillance data provides an overview of intussusception in infants in Tanzania. Similar to that reported in nearly all other countries, our age distribution had a bell-shaped curve for the first 12 months of life, with peak number of cases occurring between ages 4-7 months [1, 2, 7]. Previously published data on intussusception in children in Tanzania include a retrospective study during 2010-2012 from one hospital that also participated in this prospective evaluation; Bugando hospital [8]. Most (75%, n = 42) of their cases were in infants. Similar to our findings, only 37% of all their cases presented before day 3 of illness, and all patients were treated surgically. The overall (not only infants) case-fatality rate was 14%. That report provided additional information that we did not collect, such as abdominal distension being the most common presenting symptom (in 86%), indicating the late presentation to the referral hospital. Postoperative complications occurred in 32% of cases, with the most frequent being surgical site infection. Another of our participating hospitals, Muhimbili National Hospital had reported on pediatric intussusception cases collected retrospectively for 2000-2004, with total 21 infant cases [9]. Among all the pediatric cases, the mortality rate was 25%.

The high mortality rate for the infants cases in our series is similar to that reported on data for recent years from other countries in the continent (e.g., Rwanda, one tertiary care hospital 2009-2012: fatality rate among cases in infants = 31% (11/35); Zambia 2007-2009, fatality rate among cases aged < 2 years = 34% (31/92) [10, 11] but higher than that achieved in other regional countries (e.g., Kenya, 2002-2013, fatality rate among cases in infants = 8% [15/192]; Ethiopia, one hospital 2011-2014, fatality rate among cases aged < 13 years = 4% [6/136]; Nigeria, one hospital 2002-2011, fatality rate among pediatric cases = 5% [3/55] [12-15]. A reduction in mortality for intussusception among infants in Tanzania could be anticipated if diagnosis could be suspected and confirmed early, enema reduction was available, and facilities were strengthened in their capacity for post-operative care when surgery was still required, particularly bowel resection. These surgeons would certainly be able to perform ultrasound-guided enema reduction in cases who reach them early, if training was provided by others in the region who have the experience [16-19].

The index of suspicion for intussusception may be raised if caretaker is queried in detail (and if caretaker is able to provide such information) about the early symptoms for infants in the peak age group evaluated for repeated vomiting, perceived abdominal pain, possible dysentery, irritability or lethargy, before development of an acute abdomen. The earliest signs and symptoms of intussusception often are brief episodes of colicky abdominal pain with severe crying, often with pulling up of the legs. The baby may become pale, sweaty and lethargic during these episodes [20-22]. These episodes could come and go early on, lasting just a few minutes and might occur several times in an hour, with the baby appearing completely well between the episodes. Obtaining such a history from specific querying of the caretaker should put intussusception high on the differential and result in prompt efforts to confirm or refute the diagnosis (e.g., through abdominal ultrasound). Not all infants with intussusception have this classic history. In infants aged < 4 months, repeated vomiting may be the earliest symptom [20-22]. In bacterial dysentery, vomiting may not be a prominent component of clinical illness in infants compared with the diarrheal mucoid stools with blood [23-25] whereas in infants with intussusception vomiting is usually a prominent component of the illness history by the time blood is detected in stools. Lack of concurrent gastrointestinal illness in other household members could also raise intussusception higher on the differential. Identifying infants with intussusception early in the illness is challenging and availability and use of abdominal ultrasound is critical.

Our surveillance had limitations. We could not calculate incidence rates since hospital catchment populations could not be enumerated. Within our hospitals that participated in the surveillance, intussusception cases that arrived to the hospital but died before surgery could be performed would not have been counted in our surveillance because autopsies are not performed at these hospitals and would have been required to meet Brighton Level 1 criteria. It is also possible some other cases were missed given the challenges of enrollment. The date of symptom onset may have been difficult for parents to accurately identify if asked as a single generic question-this may possibly explain the reported symptom duration of 6-14 days for 6 children who survived even without resection. More accurate information could potentially be obtained by asking separately about onset of particular symptoms (e.g., colicky abdominal pain). As noted above, more detailed elicitation of the symptoms, if parents are able to provide the information, could increase the index of suspicion for possible intussusception. Finally, the data were collected for the self-controlled case series, and data collection was not designed to explore in detail the factors that can result in delay in receiving definitive care for intussusception nor information on post-operative complications. For example, we did not collect information on health-care seeking for the illness before first hospital admission, availability of transportation to healthcare facility, number of days from admission at first hospital to date that clinicians first suspected intussusception, availability of abdominal ultrasound at all the hospitals, and time from diagnosis to start of surgery.

## Conclusion

Our data highlight that, while intussusception is uncommon in Tanzanian infants, it has a high case-fatality rate. Raising the index of suspicion among healthcare providers for infants (especially in the peak age range of 4-7 months), having wide availability of point-of-care abdominal ultrasound for earlier diagnosis, and training teams in ultrasound-guided enema reduction used in other African countries could help reduce the fatality rate to the lower levels achieved in other countries in the region.

### What is known about this topic

- Two single-center reports were published on intussusception among Tanzanian children (not only infants) in the past decade, with 56 and 21 cases summarized;
- Late presentation was common: case-fatality rates were 14% and 25%.

### What this study adds

- This large case series (207 cases) demonstrated that intussusception among Tanzanian infants has a peak onset during ages 4 through 7 months;
- Infants with intussusception suffer a high case-fatality rate (30%);
- Prompt access to care, diagnosis and treatment are needed to reduce the high fatality rate.
